# Expression patterns of endothelial permeability pathways in the development of the blood-retinal barrier in mice

**DOI:** 10.1096/fj.201801499RRR

**Published:** 2019-01-30

**Authors:** Anne-Eva van der Wijk, Joanna Wisniewska-Kruk, Ilse M. C. Vogels, Henk A. van Veen, Wing Fung Ip, Nicole N. van der Wel, Cornelis J. F. van Noorden, Reinier O. Schlingemann, Ingeborg Klaassen

**Affiliations:** * Departments of Ophthalmology and Medical Biology, Amsterdam UMC, Ocular Angiogenesis Group, Amsterdam Cardiovascular Sciences, Cancer Center Amsterdam, University of Amsterdam, Amsterdam, The Netherlands;; † Department of Medical Biology, Amsterdam UMC, Electron Microscopy Center Amsterdam, University of Amsterdam, Amsterdam, The Netherlands;; ‡ Department of Medical Biology, Amsterdam UMC, Cellular Imaging Core Facility, University of Amsterdam, Amsterdam, The Netherlands;; § Department of Genetic Toxicology and Tumor Biology, National Institute of Biology, Ljubljana, Slovenia; and; ¶ Department of Ophthalmology, Jules Gonin Eye Hospital, University of Lausanne, Lausanne, Switzerland

**Keywords:** tight junctions, transcellular permeability, VEGF signaling

## Abstract

Insight into the molecular and cellular processes in blood-retinal barrier (BRB) development, including the contribution of paracellular and transcellular pathways, is still incomplete but may help to understand the inverse process of BRB loss in pathologic eye conditions. In this comprehensive observational study, we describe in detail the formation of the BRB at the molecular level in physiologic conditions, using mice from postnatal day (P)3 to P25. Our data indicate that immature blood vessels already have tight junctions at P5, before the formation of a functional BRB. Expression of the endothelial cell-specific protein plasmalemma vesicle-associated protein (PLVAP), which is known to be involved in transcellular transport and associated with BRB permeability, decreased during development and was absent when a functional barrier was formed. Moreover, we show that PLVAP deficiency causes a transient delay in retinal vascular development and changes in mRNA expression levels of endothelial permeability pathway proteins.—Van der Wijk, A.-E., Wisniewska-Kruk, J., Vogels, I. M. C., van Veen, H. A., Ip, W. F., van der Wel, N. N., van Noorden, C. J. F., Schlingemann, R. O., Klaassen, I. Expression patterns of endothelial permeability pathways in the development of the blood-retinal barrier in mice.

Homeostasis of the neural retina is maintained by the blood-retinal barrier (BRB), formed by the neurovascular unit consisting of endothelial cells of the retinal capillaries, pericytes, and glial cells (1, 2). In humans, the retinal vasculature develops around midgestation (3) but initially lacks a functional BRB, which is formed during further differentiation to mature endothelium. In pathologic conditions such as diabetic macular edema, the acquired BRB properties are lost in the adult retina (4), causing blindness. The molecular and cellular events in BRB development are poorly understood but may help us to understand the reverse events in pathologic BRB loss. In fact, such understanding may even enable the development of alternative treatment strategies for pathologic eye conditions associated with macular edema.

We have previously identified a specific set of genes that is associated with BRB integrity (5). The most important were genes involved in the regulation of paracellular transport (claudin-5 and occludin) and the vesicular and transcellular transport–related genes caveolin-1 and plasmalemma vesicle-associated protein (PLVAP, or PV-1). In the brain, expression of PLVAP is negatively correlated with blood-brain barrier (BBB) function (6, 7), and PLVAP protein is not detected when a functional BBB is formed (8, 9). PLVAP is also absent in mature barrier endothelium of the BRB and the testis (10, 11). Conversely, PLVAP is up-regulated in monkey retina after intravitreal injections of VEGF, a major inducer of BRB breakdown (12, 13). Moreover, increased PLVAP expression in capillaries is associated with loss of the BBB in brain tumors (7, 14) and of the BRB in human patients with diabetic retinopathy (15).

PLVAP is an endothelium-specific structural component of fenestral and stomatal diaphragms in endothelial fenestrae, caveolae, and *trans*-endothelial channels (16). The recent generation of *Plvap*-deficient mice has highlighted the structural role of PLVAP in the maintenance of size-selective permeability in fenestrated endothelia (17, 18). In fact, *Plvap*-deficient mice that survived postnatally showed growth retardation, anemia (17), and selective leakage of plasma proteins into the interstitium with subsequent edema and dyslipidemia, eventually leading to a lethal, protein-losing enteropathy (18). Crucially, a human form of PLVAP deficiency caused by a nonsense mutation in the *Plvap* gene results in a nearly identical disease profile to that observed in *Plvap*-deficient mice, characterized by hypoproteinemia, hypoalbuminemia, and hypertriglyceridemia (19). These observations indicate that in nonbarrier endothelium, PLVAP prevents excessive protein leakage into tissues, a function that is apparently not needed in a patent BBB or BRB. In fact, our group has demonstrated that in these barrier endothelia PLVAP has a reverse role, because it appears to be necessary for BRB loss and protein leakage (20). In addition, PLVAP also has a key role in physiologic and pathologic angiogenesis (unpublished results).

In the present observational study, we give a comprehensive overview of the development of the BRB in mice, with respect to paracellular and transcellular transport and VEGF signaling. Moreover, we focus on the role of PLVAP in this process, and with the use of transgenic heterozygous *Plvap* (*Plvap*^+/−^) mice, we show that PLVAP is involved in the formation of the retinal vasculature and the BRB.

## MATERIALS AND METHODS

### Generation of transgenic mice

Animal experiments were performed with the approval of the Animal Ethics Committee of the University of Amsterdam and in compliance with the Association for Research in Vision and Ophthalmology Statement for the Use of Animals in Ophthalmic and Vision Research.

*Plvap*^+/−^ mice were generated by targeting exon 1 on the *Plvap* locus by insertion of an IRES:lacZ trapping cassette and a floxed promoter-driven neo cassette, and homologous recombination in strain 129/SvEvBrd-derived embryonic stem cells. The chimeric mice were bred to C57BL/6-Tyr^c-Brd^ albino mice to generate F1 heterozygous animals. This progeny was intercrossed to generate F2 wild-type (WT), heterozygous, and homozygous mutant progeny. The B6;129S5-*Plvap^tm1LEX^*/Mmucd mice were imported from the Mutant Mouse Regional Resource Center (Davis, CA, USA) and further bred in the animal facility at the Animal Research Institute of the Academic Medical Center (Amsterdam, The Netherlands). Because complete knockout of *Plvap* results in *in utero* or perinatal mortality in the majority of cases, and the homozygous *Plvap* mice that do survive showed postnatally a very strong phenotype and died within 2–4 wk, we used *Plvap*^+/−^ mice, which have significantly decreased *Plvap* expression and no apparent systemic phenotype (17, 18).

To study the development of the BRB, neonatal WT mice were killed on postnatal days (P)3, 5, 7, 9, 11, 13, 15, 17, and 25 with an intracardial injection of ketamine-medetomidine-atropine for young mice (until P13); older mice were euthanized with CO_2_ asphyxiation. Heterozygous littermates were killed on P5, 9, 13, and 25. Eyes were enucleated and either snapfrozen in liquid nitrogen (for quantitative PCR or immunohistochemistry) or processed immediately for retinal wholemount staining.

### Genotyping

Genotyping was performed by PCR analysis, using forward and reverse primers 5′-TCCTCTTCGTGTCGCTCATTCAG-3′ and 5′-CTTACCAGGTCGCCTTGGCAC-3′, resulting in a 289 bp PCR fragment for the WT allele, and forward and reverse primers 5′-GTTGCATGTACTACACCAGG-3′ and 5′-GCAGCGCATCGCCTTCTATC-3′, resulting in a 395 bp fragment for the targeted allele. Genomic DNA was obtained from toes. Genomic DNA was isolated using the quick and dirty protocol according to Truett *et al.* (21). PCR analysis was performed with GoTaq Hot Start Green Master Mix (Promega, Madison, WI, USA) containing GoTaq Hot Start Polymerase, deoxynucleotide triphosphates, MgCl_2_, and reaction buffers in 25 µl reaction volumes. The cycling conditions consisted of hot start initiation at 94°C for 5 min, followed by denaturation for 15 s at 94°C, annealing for 30 s at 60°C, and elongation for 40 s at 72°C, for 40 cycles.

### Western blot analysis

Protein was extracted from kidney samples on ice by homogenizing the tissue in RIPA buffer (Thermo Fisher Scientific, Waltham, MA, USA) supplemented with protease inhibitors. Insoluble constituents were removed by centrifugation for 10 min at 4°C, at maximum speed. For Western blot analysis, 50 µg protein was subjected to SDS-PAGE on a precast gradient gel (Bio-Rad, Hercules, CA, USA) and transferred to a PVDF membrane (MilliporeSigma, Burlington, MA, USA) by wet blotting. After blocking for 1 h with 5% BSA in Tris-buffered saline with 0.05% Tween20 (TBS-T), membranes were incubated overnight at 4°C with rat anti–panendothelial cell antigen IgG_2a_ antibody (MECA-32; Santa Cruz Biotechnology, Dallas, TX, USA), diluted 1:500 in 5% BSA in TBS-T. After washing in TBS-T, membranes were incubated for 1 h at room temperature with rabbit anti-rat horseradish peroxidase (Agilent Technologies, Santa Clara, CA, USA), diluted 1:10,000 in 0.5% BSA in TBS-T. Membranes were washed twice in TBS-T and once in Tris-buffered saline, incubated for 5 min with chemiluminescent substrate (SuperSignal West Pico Chemiluminescent Substrate; Thermo Fisher Scientific) and visualized on an ImageQuant LAS 4000 Imager (GE Healthcare, Waukesha, WI, USA).

### RNA isolation and mRNA quantification

Retinas (at least 6–8 retinas per group) were treated by hypotonic lysis to enrich for retinal vessels (22). Each retina was incubated in 1 ml sterile water for 2 h at 4°C. Next, retinas were spun down, and sterile water was replaced with sterile water containing 40 µg DNase I (Thermo Fisher Scientific) and left for 5 min at room temperature. Retinas were spun down, supernatant was removed, and the retinal vessels were resuspended in 500 µl Trizol reagent (Thermo Fisher Scientific) and stored at −20°C until further processing. Total RNA was isolated according to the manufacturer’s protocol and dissolved in RNAse-free water. RNA yield was measured using a NanoDrop spectrophotometer (Thermo Fisher Scientific), and 1 µg of RNA was treated with DNAse-I (Thermo Fisher Scientific) and reverse transcribed into first-strand cDNA with Maxima First Strand cDNA Synthesis Kit (Thermo Fisher Scientific). Real-time quantitative PCR was performed on 20× diluted cDNA samples using a CFX96 system (Bio-Rad) as previously described (5). Specificity of the primers was confirmed by the U.S. National Center for Biotechnology Information’s Basic Local Alignment Search Tool (NCBI BLAST). The presence of a single PCR product was verified by both the presence of a single melting temperature peak and detection of a single band of the expected size on 3% agarose gel. Nontemplate controls were included to verify the method and the specificity of the primers. Relative gene expression was calculated using the equation *R* = *E*^−^*^Ct^*, where *E* is the mean efficiency of all samples for the gene being evaluated and *C_t_* is the cycle threshold for the gene as determined during real-time PCR. Primer efficiencies (*E*) were determined with LinRegPCR software (23) and ranged from 1817 to 1979. PCR products that did not show a single melting temperature peak were excluded from analysis. Expression data was normalized by the global mean normalization method using expression data of all tested genes (24).

### Retinal wholemount and cryostat section staining

Enucleated mouse eyes were washed in PBS, fixed in 4% paraformaldehyde for 5 min, and transferred to 2× PBS for 10 min; retinas were dissected in PBS. Isolated retinas were postfixed in methanol and stored in −20°C until further use. For immunofluorescent staining, retinas were briefly washed in 2× PBS and incubated in wholemount-blocking buffer (1% fetal calf serum, 3% TritonX-100, 0.5% Tween20, 0.2% sodium azide in 2× PBS) for 2 h at room temperature. Next, retinas were incubated overnight with the following antibodies: rabbit anti–claudin-5 (diluted 1:250, 34-1600; Thermo Fisher Scientific), isolectin B4 (Alexa Fluor-488 labeled, diluted 1:30, I21411; Invitrogen) or rat anti–MECA-32 (diluted 1:100, 553849; BD Biosciences, San Jose, CA, USA) diluted in wholemount blocking buffer. After 3 wash steps (3 × 30 min in wholemount blocking buffer), secondary antibody was added for 2–3 h [diluted 1:100, goat anti-rabbit Alexa Fluor-633, goat anti-rabbit cyanine 3 (Cy3), or goat anti-rat Cy3; Thermo Fisher Scientific] diluted in wholemount blocking buffer. When retinas were stained with rat anti–MECA-32, 2% normal mouse serum was added to the secondary antibody mix. After overnight washing in wholemount blocking buffer, retinas were mounted on glass slides and covered in Vectashield (Vector Laboratories, Burlingame, CA, USA). All staining procedures were performed under gentle agitation at room temperature. For staining of the cryostat sections, the samples were fixed for 10 min with 4% paraformaldehyde, permeabilized for 10 min with 0.2% Triton X-100, and blocked for 1 h with 10% normal goat serum. Staining was done in wholemount blocking buffer with rabbit anti-mouse IgG (Agilent Technologies) or rabbit anti-fibrinogen (Abcam, Cambridge, MA, USA) for 2 h, followed by secondary antibody incubation (goat anti-rabbit Cy3) for 1 h.

Images of wholemounts were taken at the central (at the site of the optic nerve head), middle, and peripheral retina and were recorded using a confocal laser scanning microscope (SP8; Leica Microsystems, Wetzlar, Germany) with a ×20 or ×63 objective at the Cellular Imaging Core Facility of the Academic Medical Center. Specificity of the staining was checked by absence of fluorescent signal in samples where primary antibody was omitted.

For quantification of vascular density, *x*-*y* sections at the superficial, intermediate, and deep layer were taken at 4 regions around the central retina (starting at the optic nerve head), and measurements of these regions were averaged per retina. Images were thresholded and binarized with Matlab (v.R2015a; MathWorks, Natick, MA, USA) from which vascular coverage was determined (ratio of black pixels to total amount of pixels).

Quantification of filopodia at P5 was performed using high quality images with 12 bits and 1024 × 1024 pixels. To determine the length of the vascular sprouting front, we designed a macro for ImageJ software (National Institutes of Health, Bethesda, MD, USA) (Supplemental Methods). Filopodia were manually counted using the multipoint tool in ImageJ. Per animal, 3–6 images were analyzed and averaged (*n* = 5 animals for WT and *n* = 7 animals for *Plvap*^+/−^), and data are presented as the number of filopodia per 100 µm vascular sprouting front.

### Modified Miles’ assay in the skin

Mice (*n* = 5/group) were anesthetized with an intraperitoneal injection of ketamine-medetomidine-atropine mix, directly followed by an intraperitoneal injection of 150 µl Evans Blue (EB) dye (30 mg/ml; MilliporeSigma). The back skin was shaved, EB dye was allowed to circulate for 20 min, and then mouse recombinant (mr) VEGF_164_ (200 ng; Sanquin, Amsterdam, The Netherlands) and histamine (500 ng; MilliporeSigma) in 25 µl PBS were injected intradermally into flank skin. PBS (25 µl) was used as negative control and injections were performed in duplicate. After 20 min, mice were euthanized with an overdose of pentobarbital (250 mg/kg), and the tissue containing extravasated EB dye was harvested with an 8 mm biopsy puncher and incubated for 18 h in 200 µl formamide at 70°C. Extracted EB was quantified using a spectrophotometer (BMG PolarStar; MTX Lab Systems, Bradenton, FL, USA) set at 650 nm. Measurements of skin were normalized to EB circulating in the bloodstream taken from a cardiac puncture. To correct for differences between the experiments, factor correction was applied using Factor Correction software v.2015.2.0.0 (25).

### Transmission electron microscopy of mouse eyes

Eyes were harvested and immersion fixed in McDowell phosphate buffer. To facilitate entry of the fixative into the eye, eyes were punctured with a 29-gauge needle, and the cornea was cut off. Samples were processed for routine transmission electron microscopy (TEM), as described in Wisniewska-Kruk *et al.* (20). Ultrathin sections of 80 nm were examined with a Technai-12 G2 Spirit Biotwin microscope (FEI, Eindhoven, The Netherlands), and micrographs were taken with a Veleta TEM camera (Emsis, Münster, Germany) using Radius acquisition software (Emsis) at a magnification of ×30,000 at the Cellular Imaging Core Facility of the Academic Medical Center. The images were quantified with iTEM software (Olympus Soft Imaging Solutions; Olympus, Tokyo, Japan). The number of caveolae in endothelial cells was manually counted on the luminal and abluminal side in retinal capillaries in the inner or outer plexiform layer and expressed per micrometers endothelial cell wall. A total of 54 capillaries were analyzed for WT mice (*n* = 2) and 49 capillaries for *Plvap*^+/−^ mice (*n* = 4).

### Statistics

Data are depicted as means ± sd. Differences between groups were tested using a Mann-Whitney *U* test for nonparametric data or ANOVA with posthoc Bonferroni’s test, where appropriate. Differences were considered statistically significant when *P* ≤ 0.05. Statistical analyses and graphing were performed using GraphPad Prism 6 (GraphPad Software, La Jolla, CA, USA) software.

## RESULTS

### PLVAP expression in the retinal vasculature decreases during BRB development

**Figure 1:**
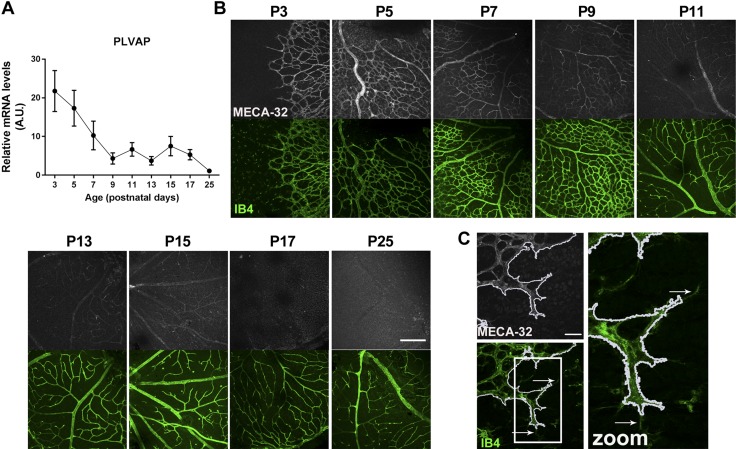
Retinal PLVAP expression decreases during BRB development. *A*) *Plvap* mRNA levels were highest at P3 in the retinal vasculature and decreased from P3 to P25; *n* = 7–11 for all time points. Data are depicted as means ± sd. *B*) Immunolocalization of PLVAP protein was visualized using MECA-32 antibody (gray) in retinal wholemounts. Isolectin B4 (IB4, green) was used to stain the retinal vessels. PLVAP expression decreased over time. Scale bar, 200 µm. *C*) PLVAP was not expressed in the filopodia of tip cells. White line outlines MECA-32 expression (outlined with magic wand tool in ImageJ), and white arrows indicate filopodia. Images are captured of a retina at P3. Scale bar, 40 µm.

During BRB development, we observed a decrease in *Plvap* mRNA levels over time, with a significant decrease from P3 to P9 and virtually no expression from P9 onwards (**Fig. 1*A***). Immunolocalization of PLVAP protein in retinal wholemounts with the MECA-32 antibody followed a pattern similar to *Plvap* mRNA transcript levels over time (Fig. 1*B*). PLVAP expression was high at P3 and P5 and decreased to absent immunoreactivity of PLVAP protein at P17 and P25. At early time points, PLVAP was expressed in all retinal vessels (*i.e.*, arteries, capillaries, and veins). However, PLVAP was absent from the filopodia of tip cells at the vascular sprouting front (Fig. 1*C*). Expression was highest in the superficial vascular plexus and lowest in the deep capillary plexus (at P9; unpublished results).

The timing of the decrease in PLVAP expression that we observed coincided with formation of a functional BRB in mice (at P10), as was recently shown by tracer studies using Sulfo-NHS-Biotin (*N*-hydroxysulfosuccinimidobiotin; 550 Da) and fluorescently labeled 10-kDa dextran (26). Here, we used immunohistochemical staining of the plasma protein IgG, an endogenous marker of protein leakage. In mature and functional barrier endothelium, plasma proteins are confined to the lumen of the vessels, whereas extravasation of plasma proteins indicates an immature or leaky barrier (12). In the first postnatal weeks, IgG was localized in and around the superficial vessels in the ganglion cell layer, indicating protein leakage (Supplemental Fig. S1). At P13, only occasional staining around vessels was observed. From P15 onwards, IgG was confined to the vessel lumen. Staining of IgG in the choriocapillaris served as a positive control of plasma protein leakage at all time points (Supplemental Fig. S1), because the choriocapillaris consists of highly permeable fenestrated endothelium (10). Together, these data indicate a negative relationship between PLVAP expression and formation of the functional BRB.

### Endothelial junction gene expression is sequentially regulated during BRB formation

**Figure 2:**
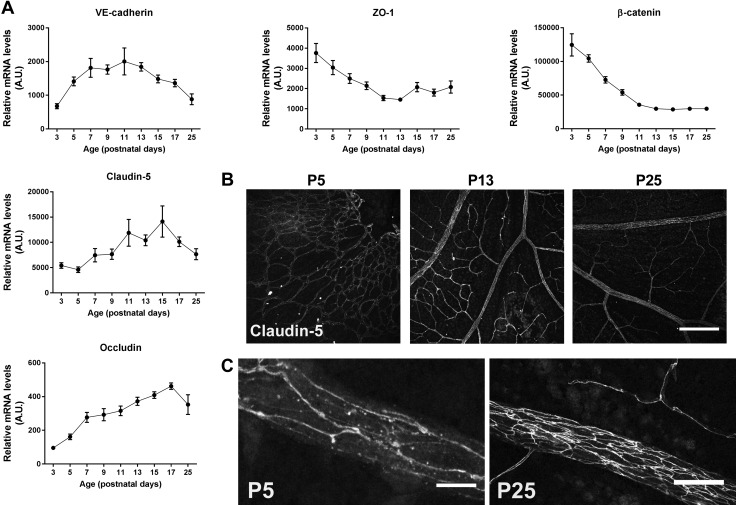
Expression of tight junctions increases during BRB development. *A*) mRNA levels of VE-cad, β-catenin, occludin, ZO-1, and claudin-5 in the retinal vasculature from P3 to P25. *n* = 7–11 for all time points. A.U., Arbitrary units. Data are depicted as means ± sd. *B*) Protein expression of claudin-5 (gray) in retinal wholemounts was present at all ages. Scale bar, 200 µm. *C*) At P5, claudin-5 (gray) was localized at the cell membrane, and its membrane expression increased at later time points. Retinal veins are shown. Scale bars, 10 µm (P5) and 50 µm (P25).

Endothelial cells of the BBB and BRB are known to form a tight barrier with limited paracellular and transcellular transport, strictly maintaining homeostasis of the neural tissues. In pathologic BRB loss, increased paracellular leakage through disruption of tight and adherens junctions is one of the suggested mechanisms (4, 27). During development, expression of VE-cadherin (VE-cad), occludin, and claudin-5 mRNA levels increased in retinal vessels from neonatal mice (**Fig. 2*A***), which may be a sign of formation of functional junctions allowing a functional BRB. VE-cad and occludin mRNA levels almost doubled from P3 to P5, and VE-cad levels increased until P11, from there it showed a marked decrease. Occludin peaked at P17, almost 5-fold higher compared with P3. Claudin-5 started to increase from P5 onwards and peaked at P15. mRNA levels of zonula occludens 1 (ZO-1) and β-catenin, the latter of which is involved in Wnt signaling but is also part of adherens junctions, showed a relative decrease over time. In a comparison of the abundance of the tight and adherens junction components (based on arbitrary units; see the Materials and Methods section), claudin-5, ZO-1, and β-catenin were already present at relatively high levels at P3, whereas VE-cad and occludin were less abundant (Fig. 2*A*). Immunostaining on cryostat sections showed expression of VE-cad, ZO-1, and claudin-5 from P5 onwards (Fig. 2*B* and Supplemental Fig. S2*A*–*D*). Moreover, staining of retinal vessels in wholemounts showed that at P5, claudin-5 protein was localized at the cell membranes and in the cytoplasm. The cell membrane localization appeared less pronounced compared with later time points (Fig. 2*C*). These data indicate that expression of tight and adherens junctions is temporally regulated and that already at P5 the immature vessels appear to have tight junctions, before a functional BRB is formed.

### Reduced PLVAP expression delays retinal vascularization at P5

**Figure 3:**
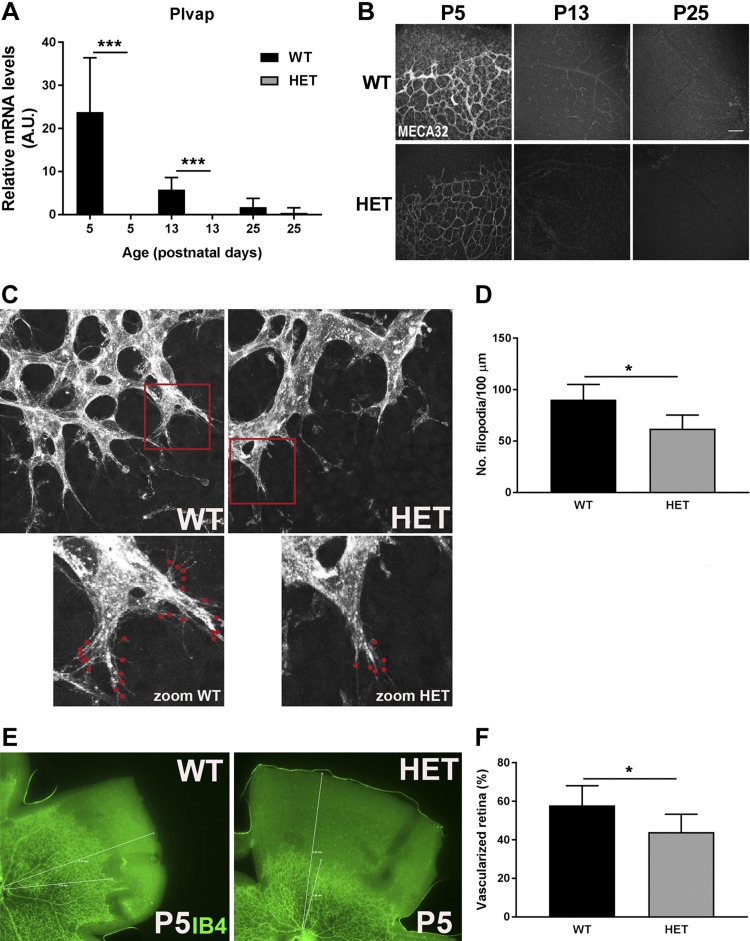
Reduced PLVAP expression delays retinal vascularization at P5. *A*) Plvap mRNA was below detection levels in retinal vasculature of *Plvap*^+/−^ mice (HET), whereas expression levels were significantly higher in WT mice at P5 and P13. *B*) PLVAP protein expression was visualized in retinal wholemounts of WT and HET mice using MECA-32 (gray) at P5, 13, and 25. There is protein expression of PLVAP in HET retina at P5, although it is reduced compared with WT retina. Scale bar, 100 µm. *C*) Sprouting endothelial cells in the retina of HET mice have less filopodia compared with WT mice. Red dots indicate filopodia in the vascular sprouting front. *D*) Quantification of the number of filopodia per 100 µm vascular sprouting front was performed using ImageJ, as indicated in the Materials and Methods section; *n* = 5 for WT and *n* = 7 for HET. *E*) Retinal vascularization was imaged in retinal wholemounts of WT and HET mice using isolectin B4 (IB4, green) at P5, which was delayed in HET mice. *F*) Quantification of retinal vascularization at P5; *n* = 6 for WT and HET. Data are depicted as means ± sd; *P < 0.05, ***P < 0.01.

To investigate a possible role of PLVAP in barrier endothelium, we used transgenic *Plvap* mice. To confirm that *Plvap*^+/−^ mice have less PLVAP, we checked mRNA and protein levels in kidneys and observed significantly reduced PLVAP expression in *Plvap*^+/−^ mice compared with WT mice (Supplemental Fig. S3*A*–*C*). In the retina, *Plvap* mRNA levels were below the detection limit in the retinal vasculature of *Plvap*^+/−^ mice (**Fig. 3*A***), whereas PLVAP protein was present at P5 (albeit in reduced levels compared with WT mice), but not at P13 and P25 (Fig. 3*B*). Because we have recently observed that decreased expression of PLVAP leads to reduced angiogenesis and reduced endothelial migration, sprouting, and tip cell numbers *in vitro* (unpublished results), we assessed in the present study whether PLVAP is necessary for the formation of the vascular network in the retina by comparing retinal vascularization at P5, when the superficial retinal vasculature is still extending from the central retina to the periphery, in WT and *Plvap*^+/−^ mice. We found that sprouting endothelial cells in the retina in *Plvap*^+/−^ mice have less filopodia at P5 compared with those in WT controls (Fig. 3*C, D*). In addition, retinal vascularization was delayed in *Plvap*^+/−^ mice at P5, with 58.0 ± 10.1% of the retina vascularized in WT animals *vs.* 44.0 ± 9.3% in *Plvap*^+/−^ mice (*P* < 0.05; Fig. 3*E, F*).

### Reduced PLVAP expression does not affect retinal vascular density at later time points

**Figure 4:**
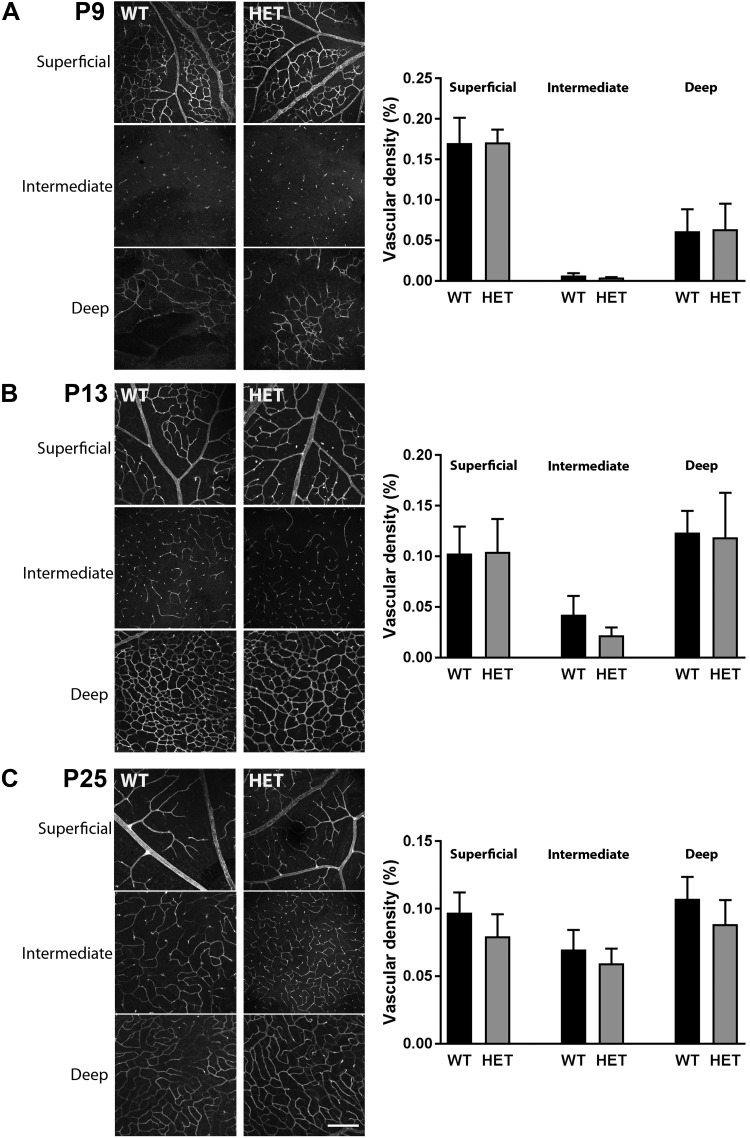
Reduced PLVAP expression does not alter vascular density. Vascular density of the retina was visualized with isolectin B4 in WT and *Plvap*^+/−^ mice (HET) at P9 (*A*), P13 (*B*), and P25 (*C*) (left panels) and quantified in the central retina (right panels), using Matlab software; *n* = 3–5 per time point for HET and *n* = 4–8 per time point for WT. Scale bar, 200 µm. Data are depicted as means ± sd.

We selected 3 crucial time points to compare further development of vascularization between WT and *Plvap^+/−^* mice: P9, when the deep capillary layer is being formed, P13, when the deep capillary layer is complete and the intermediate layer is being formed, and P25, when the formation of all 3 vascular plexi is complete. Despite the aforementioned vascularization delay at P5, vascularization of the *Plvap*^+/−^ retina followed essentially the same pattern as that of WT mice, with a fully vascularized retina at P25 (**Fig. 4**). Quantification in the central retina showed that vascular density of the superficial, intermediate, and deep layers was not affected by reduced PLVAP expression at P9, 13, and 25 (Fig. 4). The diameters of arteries and veins of the central retina were also not affected in *Plvap^+/−^* mice (unpublished results). However, a closer examination of the fully vascularized retinas (≥P25) revealed that, instead of capillaries branching off of veins in the superficial vascular plexus to form the parallel intermediate and deep capillary layers, the retinal vein itself traversed across the different retinal layers in a number of eyes, ending in the deep capillary layer. This was more often the case in *Plvap*^+/−^ mice (20.6% of veins, 2.7% of arteries) than in WT mice (9.8% of veins, 3.3% of arteries; Supplemental Table S1 and Supplemental Videos S1 and S2). Taken together, these data indicate that reduced PLVAP expression during development does not ultimately affect vascular density in the retina but does have a temporary effect on vascular growth and on the spatial distribution of veins in the fully developed retina.

### Reduced PLVAP expression affects mRNA levels of retinal tight junction proteins

**Figure 5:**
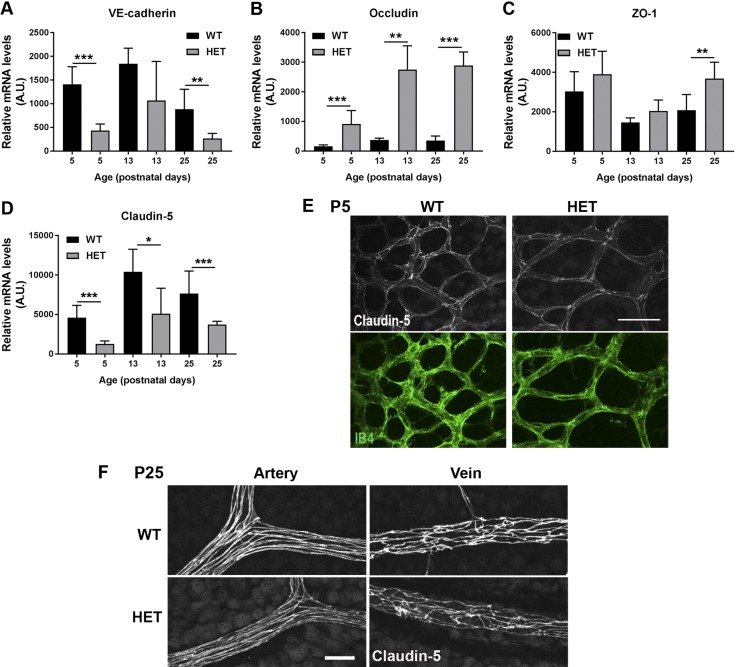
Reduced PLVAP expression affects retinal tight junctions. *A*–*D*) mRNA levels of VE-cad (*A*), occludin (*B*), ZO-1 (*C*), and claudin-5 (*D*) in the retinal vasculature in WT and *Plvap^+/−^* (HET) mice at P5, P13, and P25; *n* = 7–8 for both groups at each time point. Data are depicted as means ± sd. *P < 0.05, **P < 0.01, ***P < 0.001. *E*, *F*) Protein expression of claudin-5 (gray) in retinal wholemounts of WT and HET mice at P5 (*E*) and P25 (*F*). A.U., Arbitrary units. Scale bars, 50 µm.

To determine whether reduced PLVAP expression during development affects BRB formation, we compared expression of BRB-specific components of WT and *Plvap*^+/−^ mice at P5, 13, and 25. At these time points, the mRNA expression of tight and adherens junction genes followed the same pattern in WT and *Plvap*^+/−^ mice over time [*e.g.*, VE-cad increased from P5 to P13 but then decreased again between P13 and P25, and occludin expression increased (**Fig. 5*A–D***)]. However, in a comparison of the relative mRNA levels with WT mice, VE-cad mRNA levels were lower in *Plvap*^+/−^ mice at all time points (Fig. 5*A*), whereas differences where not found in β-catenin expression levels between WT and *Plvap*^+/−^ mice (unpublished results). In contrast, occludin mRNA levels at all 3 time points, and ZO-1 at P25, were higher in *Plvap*^+/−^ mice (Fig. 5*B, C*). Although claudin-5 mRNA levels were lower at all time points in *Plvap*^+/−^ mice (Fig. 5*D*), protein expression of claudin-5 at P5 and P25 did not appear much different (Fig. 5*E, F*). These data indicate that PLVAP directly or indirectly affects gene expression of endothelial junction proteins.

### Reduced PLVAP expression affects BRB transport mechanisms

**Figure 6:**
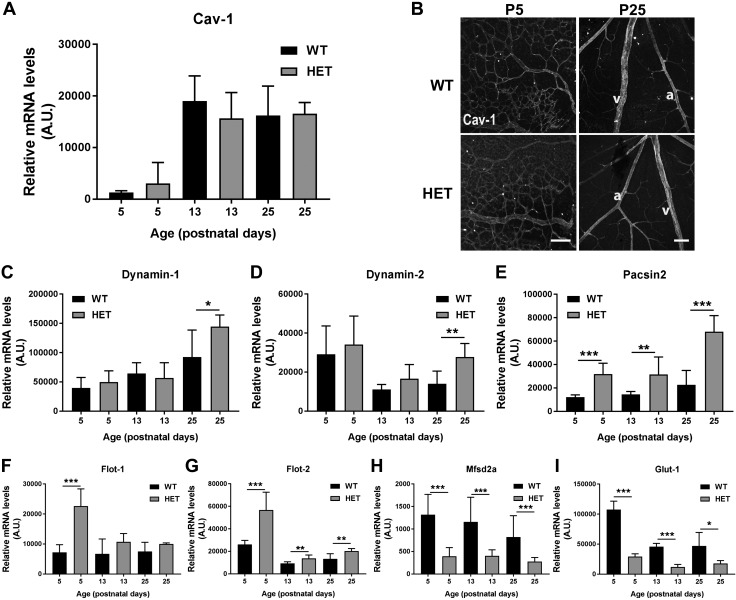
Reduced PLVAP expression affects BRB transport mechanisms. *A*) mRNA levels of caveolin-1 (cav-1) in the retinal vasculature of *Plvap*^+/−^ (HET) and WT mice. *B*) Caveolin-1 protein expression (gray) was similar in retinal wholemounts of HET and WT mice at P5 and P25. A, artery; v, vein. Scale bars, 100 µm. *C–I*) mRNA levels of dynamin-1 (*C*), dynamin-2 (*D*), pacsin-2 (*E*), Flot1 (*F*), Flot2 (*G*), Mfsd2a (*H*), and Glut-1 (*I*) in the retinal vasculature of WT and HET mice of P5, 13, and 25; *n* = 7–8 for both groups at each time point. A.U., Arbitrary units. Data are depicted as means ± sd. *P < 0.05, **P < 0.01, ***P < 0.001.

PLVAP is a structural component of caveolae (16) and is involved in transcellular transport (20). The levels of caveolin-1, the primary structural protein of caveolae (28), increased over time in the retinal vasculature, but levels were similar in *Plvap*^+/−^ and WT mice, at both the mRNA (**Fig. 6*A***) and protein level (Fig. 6*B*). However, several other proteins involved in transcellular (caveolar) transport were increased in *Plvap*^+/−^ mice. Dynamin-1 and -2, both essential for the fission of caveolae from the plasma membrane to form free transport vesicles (29), and Pacsin2, involved in the formation of caveolae (30), were increased in *Plvap*^+/−^ mice at P25 (Fig. 6*C–E*). Expression of flotillin-1 and -2 (Flot1 and -2, respectively), 2 membrane-associated proteins involved in endocytosis that are expressed in the mouse retina (5, 31), also showed higher mRNA expression at P5 (*Flot1*) or at all time points (*Flot2*), respectively, compared with WT mice (Fig. 6*F, G*). In contrast, *Msfd2a*, a known suppressor of transcytosis in BBB and BRB formation (26, 32), was significantly less expressed in *Plvap*^+/−^ mice at all time points (Fig. 6*H*). In addition, mRNA levels of the glucose transporter *Glut1* [an indicator of BBB function and development (33)], were significantly lower in *Plvap*^+/−^ mice at all time points (Fig. 6*I*). Although the above results suggest a possibly increased transcellular transport, perivascular IgG staining in retinal cryostat sections was similar in WT and *Plvap*^+/−^ mice at P5 and P25 (unpublished results). This was confirmed in the adult brain, where IgG staining patterns were similar in WT and *Plvap^+/−^* mice (P55; Supplemental Fig. S4). Together, these data indicate that decreased PLVAP levels during development lead to altered expression of proteins involved in (caveolar) transcellular transport, but this does not appear to be associated with overt functional changes in protein permeability, as was shown by IgG leakage patterns.

### PLVAP expression does not affect the number of caveolae in the retina

**Figure 7:**
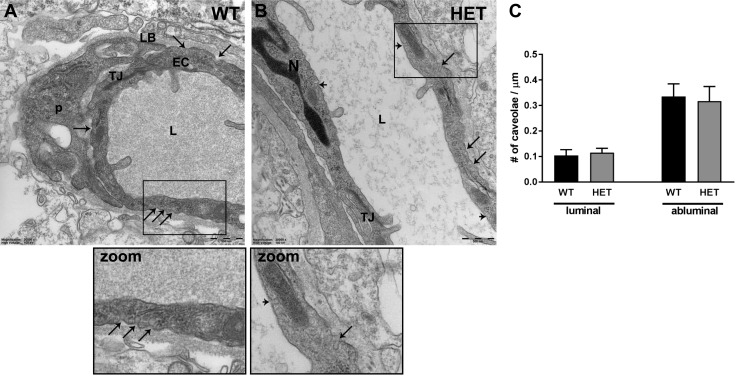
PLVAP expression does not affect the number of caveolae in the retina. *A*, *B*) Caveolae were observed at the abluminal and luminal side of endothelial cells in WT (*A*) and *Plvap*^+/−^ (HET) (*B*) mice at P30, as shown at the ultrastructural level with TEM. Scale bars, 500 nm. *C*) Quantification of the number of caveolae/µm in retinal capillaries at the luminal and abluminal side in WT and HET mice. EC, endothelial cell; L, lumen; LB, lamina basalis; N, nucleus; P, perivascular cell; TJ, tight junction; large arrow, abluminal caveolae; small arrow, luminal caveolae. Data are depicted as means ± sd.

Given the changes in components of caveolae-dependent transport in *Plvap*^+/−^ mice, we compared retinal capillaries in the inner and outer plexiform layer of WT and *Plvap*^+/−^ mice at the ultrastructural level. In human retinal explants, we previously showed that knockdown of PLVAP expression blocked the formation of caveolae after VEGF stimulation whereas basal levels of caveolae in endothelial cells were not affected (20). In line with this, we did not find any differences in the number of abluminal or luminal caveolae between WT and *Plvap*^+/−^ mice (**Fig. 7**). In both groups, only few endothelial caveolae were found, and were located more frequently on the abluminal side compared with the luminal side (Fig. 7*C*), as previously reported (12).

### VEGF receptor expression during retinal vascularization

VEGF signaling is a main driver of angiogenesis during development and of neovascularization in pathologic conditions, such as proliferative diabetic retinopathy (1). PLVAP expression is regulated by VEGF in a VEGF receptor 2 (VEGFR2)–dependent manner (34). In developing retinal vessels of WT mice, we found similar patterns of mRNA levels of VEGFR2 (*Kdr*) and its coreceptors *Nrp1* and *Nrp2*, with high expression at P3 and a decrease over time (Supplemental Fig. S5*B*, *D*, *E*), and this pattern matched the pattern of *Plvap* expression (Fig. 1*A*). In contrast, VEGFR1 (*Flt1*) expression was low at P3 but showed a steep increase over time, with high expression at P25 (Supplemental Fig. S5*A*). VEGFR3 (*Flt4*) expression was more capricious, starting low at P3 but increasing erratically over time (Supplemental Fig. S5*C*). mRNA levels of *Vegfa* started off high and decreased over time in the retinal vasculature of WT mice (Supplemental Fig. S5*F*). The expression patterns of VEGFR1, -2, -3, *Nrp1* and *-2*, and *Vegfa* were similar in *Plvap*^+/−^ and WT mice (Supplemental Fig. S5). However, at P25, *Vegfa* expression was significantly higher in retinal vessels of *Plvap*^+/−^ mice, whereas VEGFR2 and -3 (at P5 and P13) and *Nrp1* expression (at all time points) were lower. This suggests that during development, VEGF signaling may be different in *Plvap*^+/−^ mice compared with WT mice.

### Reduced PLVAP levels protect against VEGF- and histamine-induced vascular leakage in continuous endothelium

**Figure 8:**
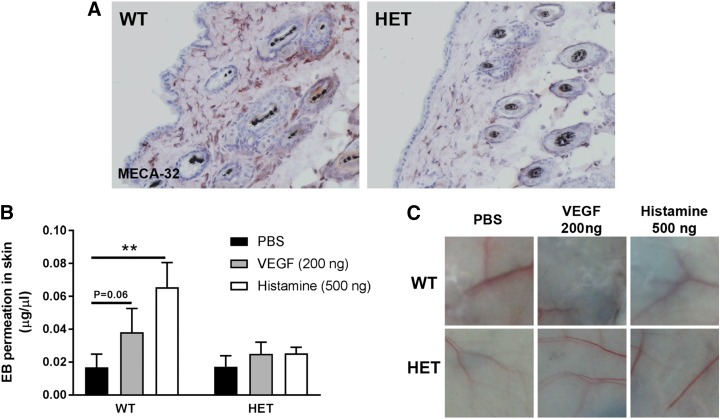
Reduced PLVAP levels protect against VEGF- and histamine-induced vascular leakage. A modified Miles assay was performed in the dorsal skin of *Plvap*^+/−^ (HET, *n* = 5) and WT (*n* = 5) mice treated with VEGF (200 ng), histamine (500 ng), and PBS as a control. *A*) MECA-32 was used to stain PLVAP in the flank vessels of WT and HET mice, showing that HET mice have reduced PLVAP expression (brown color) compared with WT mice. *B*) In WT mice, histamine and VEGF caused increased extravasation of EB, which was not the case in HET mice. Data are depicted as means ± sd. **P < 0.01. *C*) Representative images of skin injected with PBS, VEGF, or histamine in WT and HET mice.

Previously, we have shown that knockdown of PLVAP prevents both VEGF-induced permeability in an *in vitro* model of the BRB and hypoxia-induced retinal vascular leakage in an *in vivo* mouse model (20). To assess whether reduced PLVAP expression (**Fig. 8*A***) protects *Plvap*^+/−^ mice from VEGF-induced vascular leakage, we performed a modified Miles assay using the dorsal skin, which has continuous, nonfenestrated endothelium, containing caveolae with stomatal diaphragms (17). EB extravasation under basal (PBS-injected) conditions was similar in WT and *Plvap*^+/−^ mice (Fig. 8*B, C*). Histamine, a known inducer of vascular leakage (35) but not of PLVAP expression, was used as a positive control. In WT mice, VEGF (200 ng) and histamine (500 ng) both caused increased extravasation of EB, although this increase did not reach statistical significance for VEGF (*P* = 0.06). VEGF injections did not result in any increased vascular leakage in *Plvap*^+/−^ mice (Fig. 8*B, C*), providing complementary support for our previous results showing that in a BRB model PLVAP is necessary for VEGF-induced vascular leakage (20). Histamine-induced vascular leakage was also decreased in *Plvap*^+/−^ mice (Fig. 8*B, C*), indicating that PLVAP may also be downstream of histamine signaling and that PLVAP is necessary for histamine-induced leakage.

## DISCUSSION

Here, we conducted a comprehensive study using neonatal mice from P3 to P25 to describe all aspects of BRB development, with a special focus on PLVAP. We show that endothelial junction proteins of the paracellular transport pathway are expressed early, before the formation of a functional barrier. In contrast, transcellular pathway proteins like PLVAP are more closely linked to barrier function. PLVAP expression decreases during postnatal development, and its absence coincides with signs of a functional barrier. Furthermore, transcriptional levels of several BRB genes in both paracellular and transcellular endothelial transport pathways and that of VEGF signaling are affected in *Plvap*^+/−^ mice. Whether this also results in functional changes needs to be explored. We also show that PLVAP is necessary for retinal vascularization during early development in these *Plvap*^+/−^ mice, because reduced PLVAP levels caused a delay in vascularization at P5. Our study is largely observational and descriptive in nature. However, because our knowledge of cellular and molecular development of the BRB is still incomplete, we provide here a basis for further studies.

The gradual decrease of PLVAP expression from P3 to P9, with virtually no expression at P9, coincides with the formation of a functional BRB at P10 by gradual suppression of transcytosis, as recently reported (26). We used perivascular staining of IgG in cryostat sections as a marker for vascular permeability. Although diffuse staining for IgG was absent after P11, occasional staining outside the blood vessels was present at later time points (until P13). Our results may differ from those of Chow and Gu (26) because of the indirect measurements in terminated animals or other technical reasons.

As far as we know, this is the first study to follow the time course of PLVAP and BRB gene expression in detail during BRB development in mice. PLVAP is a marker of immature or leaky retinal vessels and, in addition to VEGF, is transcriptionally regulated by canonical Wnt and β-catenin signaling during development (36). When different components of the Wnt signaling pathway are inactivated (*e.g.*, Wnt-receptor ligands, receptors, or coreceptors), *Plvap* is up-regulated and vessels become leaky, also in adult mice (37). In these studies, PLVAP was expressed in WT mice at P7 and P8 (38, 39) but not at P15 (40). Using mice from P3 to P25, our study confirms and expands these observations, showing a gradual decrease in *Plvap* expression. Expression of β-catenin mRNA also decreased during development, but, considering the dual role of β-catenin in endothelium (in adherens junctions and in Wnt signaling), we cannot conclude anything about β-catenin mRNA levels in relation to PLVAP expression. PLVAP protein expression was found in all retinal vessels (arteries, capillaries, and veins), which is in agreement with an earlier report using the monoclonal MECA-32 antibody in mouse brain tissue (6). Because the MECA-32 antibody specifically recognizes murine PLVAP, these results contrast with other studies, because antibodies against human PLVAP (PAL-E, 174/2) do not stain arterioles, arteries, and large veins (41, 42).

Taking a closer look at the maturation of the BRB, we found that mRNA levels of the tight junction genes claudin-5 and occludin increased over time and stabilized in the third postnatal week, confirming previous studies (43). In contrast, ZO-1 and β-catenin showed a decrease over time, whereas VE-cad peaked around P11 and then decreased as well. This points toward temporal differences in the recruitment of different components of the tight and adherens junctions. Indeed, it was reported that adherens junctions precede tight junctions at intercellular contacts and that adherens junctions influence tight junction organization (44). Protein expression of claudin-5 was already clearly present and localized at the cell membranes of P5 and onwards. This suggests that endothelium of immature vessels already possesses tight junctions, confirming 2 recent reports that showed not only that angiogenesis occurs simultaneously with barrier genesis in the BBB (33) but also that the tight junctions in retinal vessels in mice are functional as early as P1 (26).

The generation of *Plvap*-deficient mice has allowed 2 independent research groups to unravel the functions of PLVAP in fenestrated and continuous endothelia of peripheral organs (17, 18). However, in mature barrier endothelium, PLVAP is normally absent, except in pathologic conditions like diabetic retinopathy (15), brain tumors (7, 14), and ischemic brain tissue (45, 46). Using *Plvap*^+/−^ mice, we tried to elucidate the role of PLVAP in barrier endothelium. Our data suggest that in addition to its role in transcellular permeability, PLVAP may also affect paracellular permeability *via* effects on endothelial junctions. Expression levels of ZO-1 and particularly occludin were higher in *Plvap*^+/−^ animals, whereas claudin-5 and VE-cad were lower, although this was not apparent for claudin-5 protein expression as studied in retinal wholemounts. Whether these differences are caused by direct or indirect effects of reduced PLVAP expression requires further investigation. However, in our previous studies *in vitro*, we observed a modest beneficial effect of *Plvap* inhibition on junctions of bovine retinal endothelial cells but no differences in permeability to a small molecular tracer (20), making a direct effect of PLVAP on paracellular permeability less likely.

By immunostaining of endogenous markers of protein leakage in retinal cryostat sections, we found signs of extravasation of plasma proteins during the first postnatal weeks, whereas the barrier was closed at P15 in WT mice. These observations support our hypothesis that the absence of PLVAP is a prerequisite for the BRB to be functional, because PLVAP protein expression was still present from P3 to P15 but virtually absent at later time points. However, reduced PLVAP levels during development in *Plvap*^+/−^ mice had some unexpected effects on the expression of components of the transcellular transport pathway. *Plvap*^+/−^ mice had increased mRNA expression of *Flot1*, *Flot2*, *Pacsin2*, and dynamin-1 and -2, all involved in caveolar transport. In addition, expression of *Mfsd2a*, a recently described suppressor of transcytosis in the BBB (32) and BRB (26), was significantly down-regulated in *Plvap*^+/−^ mice, suggesting that these mice may have impaired inhibition of transcytosis. Nevertheless, expression of caveolin-1, the key structural protein of caveolae, was similar in WT and *Plvap*^+/−^ retinal vessels, as was the number of endothelial caveolae. In addition, large differences in IgG extravasation were not observed between WT and *Plvap*^+/−^ mice. In fact, in the few homozygous knockout (*Plvap*^−/−^) mice that we were able to obtain, we also did not find leakage of IgG from the retinal vessels at P15. This suggests that despite the increased expression of the vesicular transcellular transport machinery and down-regulation of *Mfsd2a* expression, there is no functional increase in transcytosis under basal conditions in the retinal vessels of these mice lacking PLVAP. Considering these data and the fact that the absence, rather than the presence, of PLVAP is needed for a functional BRB, it seems that the course of barrier formation in *Plvap*^+/−^ mice may differ from the WT situation but that the final result is the same (*i.e.*, a highly restrictive barrier with very limited transport). However, it should be noted that staining for serum proteins in fixed retinal sections is suboptimal, because it does not cover leakage in the entire retina. Therefore, it would be interesting to confirm our results of BRB permeability status with other functional assays, like EB tracer or horseradish peroxidase tracer extravasation combined with EM in future experiments, to study this aspect in more detail.

Although we did not observe differences in basal retinal permeability between WT and *Plvap*^+/−^ mice, we have shown before that knockdown of PLVAP prevented hypoxia- and VEGF-driven increases in retinal vascular permeability (20). In contrast, knockdown of PLVAP in fenestrated endothelium caused a size-selective increase in permeability but did not appear to have an effect on permeability in continuous endothelia (17, 18). In line with this, we did not observe differences in basal permeability between WT and *Plvap*^+/−^ mice in continuous endothelium of the dorsal skin. However, reduced PLVAP levels prevented VEGF- and histamine-induced increases in permeability. Thus, the role of PLVAP in basal permeability may be more structural (acting as a size-selective molecular “sieve”), whereas in pathologic (VEGF- or hypoxia-induced) permeability conditions, the role of PLVAP may have an additional more active part (*e.g.*, by enhancing the VEGF signal to the endothelium). Alternatively, lack of PLVAP could render continuous endothelium with a more barrier-like endothelium status, limiting permeability to a minimum. This is in line with our previous findings, in which human umbilical vein endothelial cells had increased *trans*-endothelial electric resistance and decreased permeability after PLVAP knockdown (20).

Our group and others (45, 46) have found strong indications that PLVAP is not only a marker of vascular permeability but is also actively involved in angiogenesis. In the present study, we observed that vascularization of *Plvap*^+/−^ retinas was delayed during early development at P5, whereas the vasculature was normalized at P13. We were able to obtain a retinal wholemount and frozen tissue of 1 *Plvap*^−/−^ mouse at P5, showing an even more severe delay in vascularization (Supplemental Fig. S6*A*, *B*; *n* = 1 for *Plvap*^−/−^). However, to our surprise, even in *Plvap*^−/−^ mice, the retinal vasculature at P15 and P32 was more or less similar to that in WT and *Plvap*^+/−^ mice (Supplemental Fig. S6*C*–*E*; *n* = 1 for *Plvap*^−/−^ for both time points). Based on these findings, we conclude that PLVAP is necessary for initial retinal developmental angiogenesis but that compensatory mechanisms eventually ensure development of a normal vasculature. This is corroborated by the fact that we did not find significant differences between WT and *Plvap*^+/−^ mice when quantifying vessel density at time points beyond P5.

In the retina, development of the superficial vascular plexus is driven by a physiologic hypoxia-driven up-regulation of VEGF (47). Moreover, migration of angiogenic sprouts is dependent on signaling *via* VEGFR2, located on the tip cell filopodia (48), and VEGFR2 expression in activated endothelium correlates strongly with expression of VEGF-A (49). In the current study, we found that VEGFR1 expression increased over time, whereas VEGFR2 was initially high but decreased and stabilized with low expression at later time points. These data are in line with a report showing that in human eyes and PBS-injected monkey eyes, retinal vessels stained positively for VEGFR1, but expression of VEGFR2 and -3 was low or absent (50, 51), and indicates that in quiescent retinal endothelium there is constitutive expression of VEGFR1 but not of VEGFR2 and -3. In contrast, in diabetic human eyes with retinopathy and in VEGF-injected monkey eyes, vascular expression of all 3 VEGFRs was observed, and VEGFR2 expression coincided with PLVAP expression in the leaky retinal microvessels (50). We have found recently that PLVAP directly or indirectly regulates VEGFR2 transcription (unpublished results). Here, we again observed that VEGFR2 expression in the retinal vasculature followed the same expression pattern as *Plvap* and as expression of its coreceptors *Nrp1* and *Nrp2*. Only at P5, we found that VEGFR2 transcript levels were decreased in *Plvap*^+/−^ retinas. This was also the only time point at which we observed a delay in retinal vascularization in *Plvap*^+/−^ mice. These observations suggest that during early retinal vascular development, reduced PLVAP levels result in diminished angiogenesis, possibly *via* reduced VEGFR2-mediated signaling. At later time points, this defect is overcome and vascularization proceeds normally, despite some resulting venous anomalies that we report here. The higher incidence of aberrant retinal veins, traversing from the ganglion cell layer all the way to the outer plexiform layer, may also be related to defective VEGF-signaling in *Plvap*^+/−^ mice (*e.g.,* by not conveying the cues to halt migration and growth of endothelial sprouts at the right location). Future studies are needed to confirm this link between PLVAP levels and VEGF signaling.

In summary, in this comprehensive study we show that during BRB formation, formation of tight and adherens junctions is temporally regulated and that immature vessels without a functional barrier already have tight junctions. In contrast, functional barrier formation coincides more closely with expression patterns of the transcellular transport pathway such as PLVAP. In addition, we further characterized the role of PLVAP in developing barrier endothelium by demonstrating, with the use of *Plvap*^+/−^ mice, that PLVAP is essential in the retina during early development for proper retinal vascularization. These data confirm a role for PLVAP in angiogenesis, possibly mediated by VEGF signaling. Moreover, we suggest that in differentiated barrier endothelium, the absence of PLVAP appears to be a prerequisite for a functional BRB, because there is consistent retinal vascular leakage at times when PLVAP expression is high, and PLVAP is absent when the barrier is intact. Taken together, our data indicate that PLVAP expression may be permissive for vascular leakage as part of the transcytosis machinery, rather than having a regulatory role.

## Supplementary Material

This article includes supplemental data. Please visit *http://www.fasebj.org* to obtain this information.

## Abbreviations

BBB: blood-brain barrier
BRB: blood-retinal barrier
Cy3: cyanine 3
EB: Evans Blue
Flot1: flotillin-1
Flot2: flotillin-2
MECA-32: antipanendothelial cell antigen IgG_2a_ antibody
PLVAP: plasmalemma vesicle-associated protein
*Plvap*^+/−^: transgenic heterozygous *Plvap*
*Plvap*^−/−^: homozygous knockout *Plvap*
TBS-T: tris-buffered saline with 0.05% Tween20
TEM: transmission electron microscopy
VE-cad: VE-cadherin
VEGFR: VEGF receptor
WT: wild type
ZO-1: zona occludens 1
