# Supplementary figures and images for: Expression patterns of endothelial permeability pathways in the development of the blood-retinal barrier in mice
Source: FASEB J. 2019 Jan 30;33(4):5320–33. doi: 10.1096/fj.201801499RRR (PMC6436651; doi:10.1096/fj.201801499RRR)

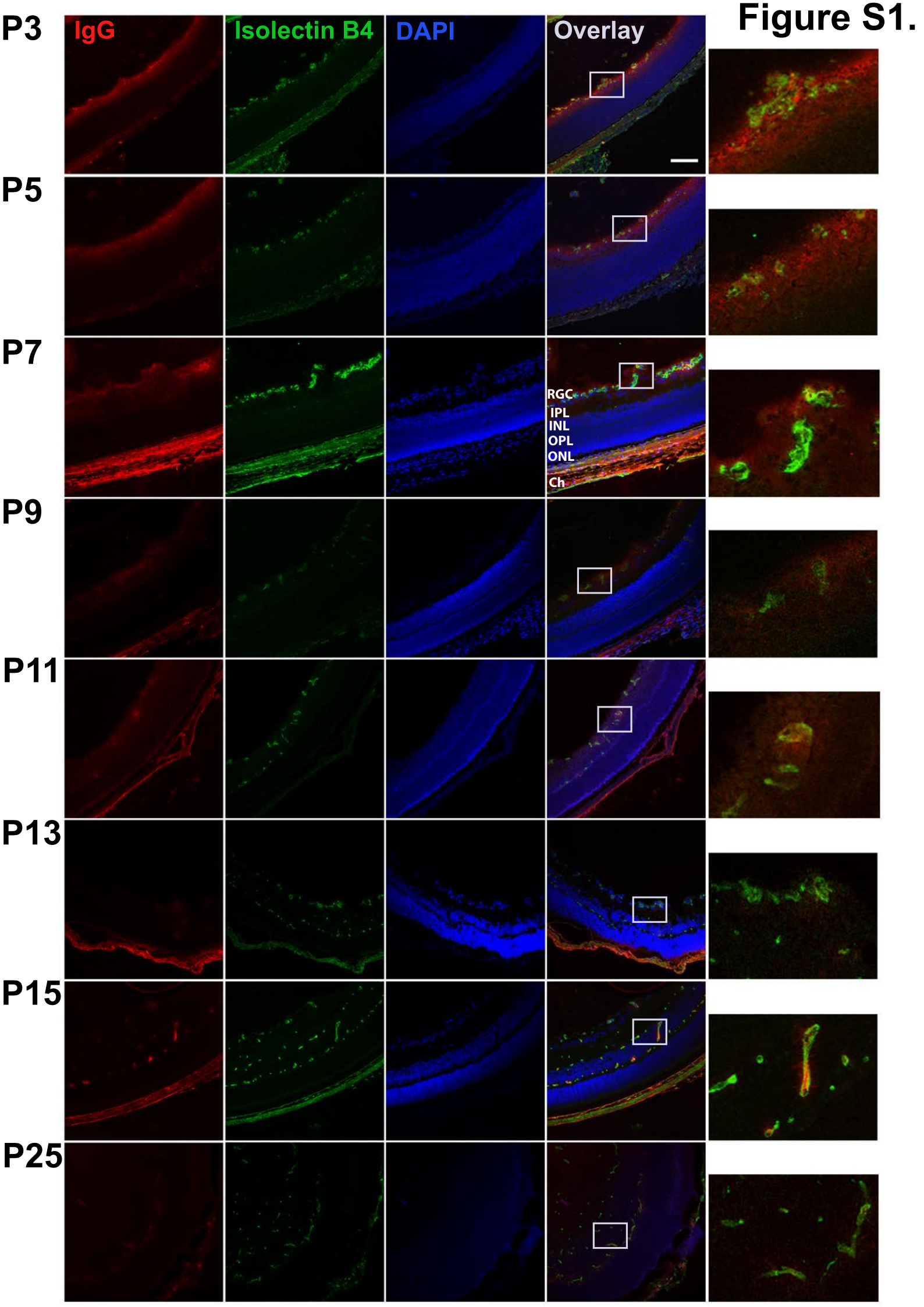

Supplement: Supplementary file 1 [file fj.201801499RRR.sf1.pdf]

**Figure S2.**

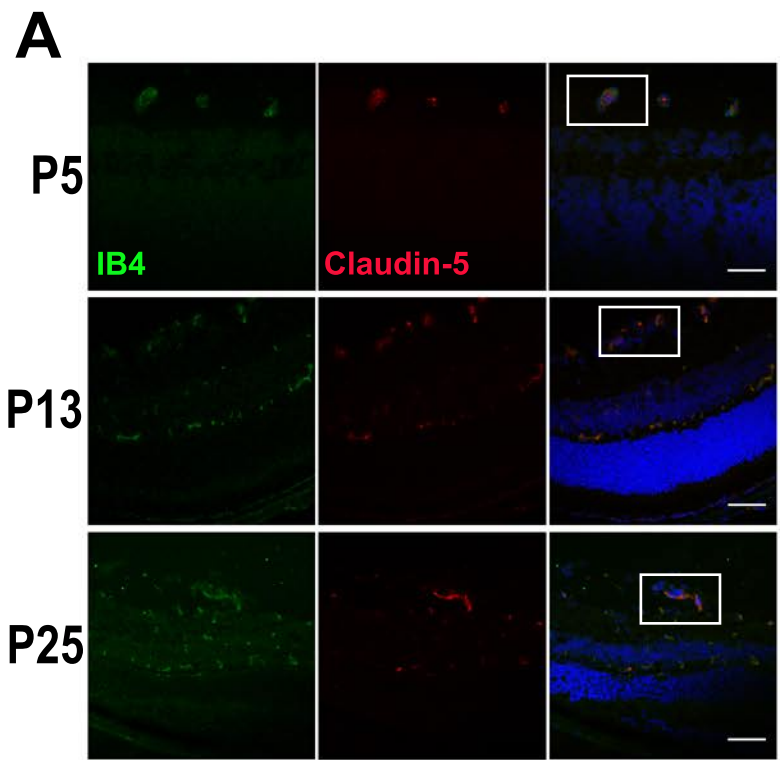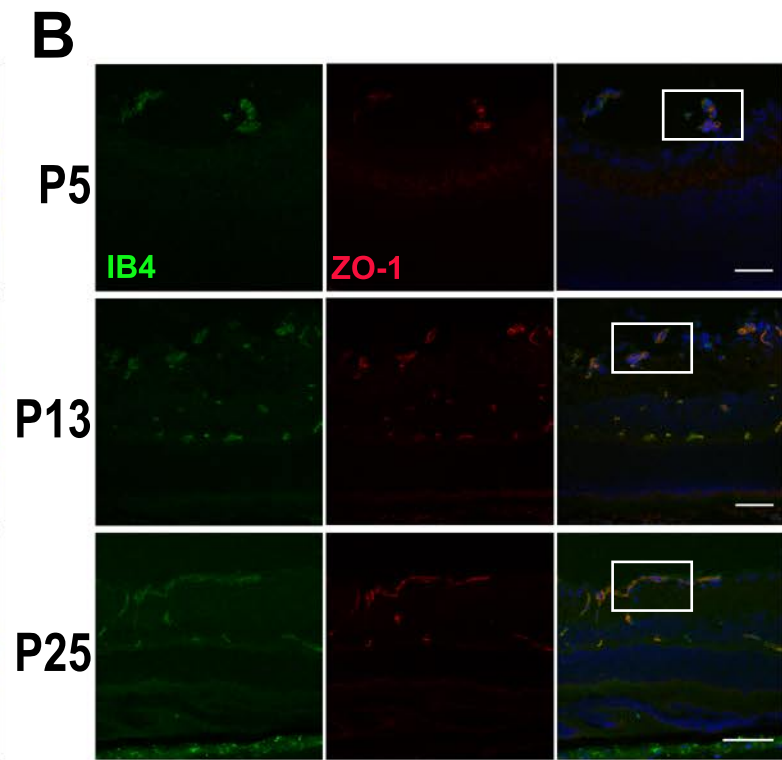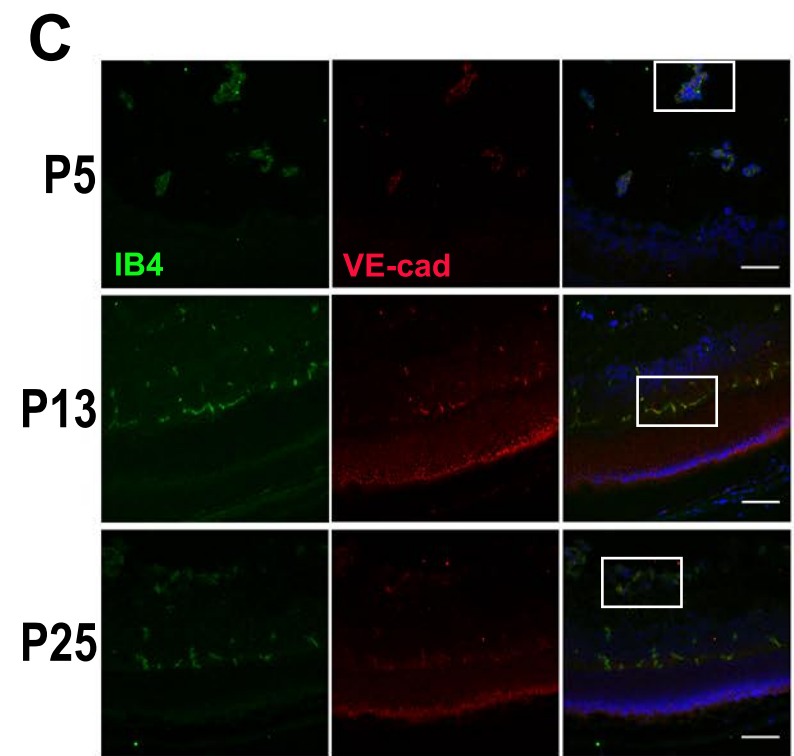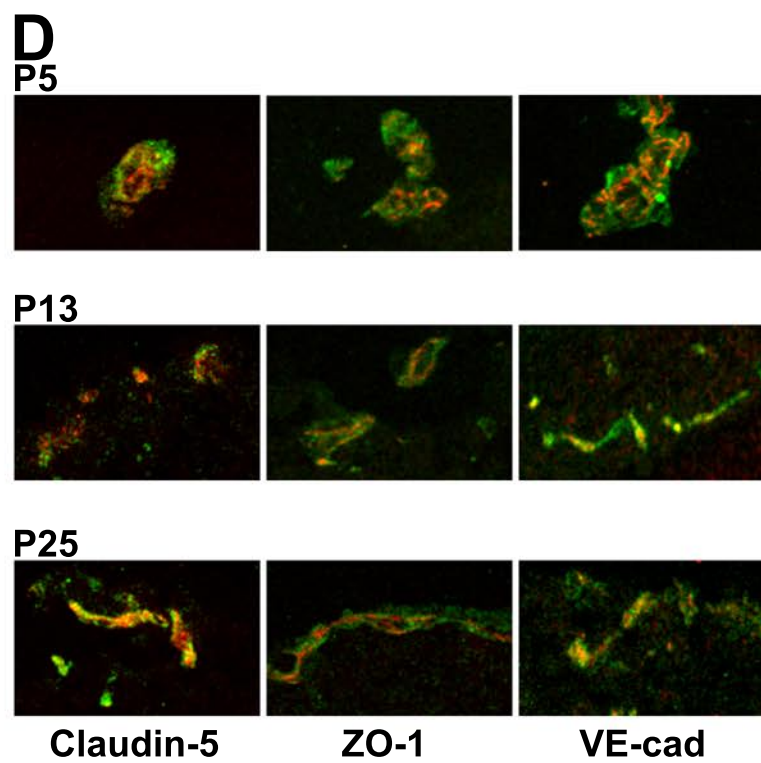

Supplement: Supplementary file 2 [file fj.201801499RRR.sf2.pdf]

**Figure S3.**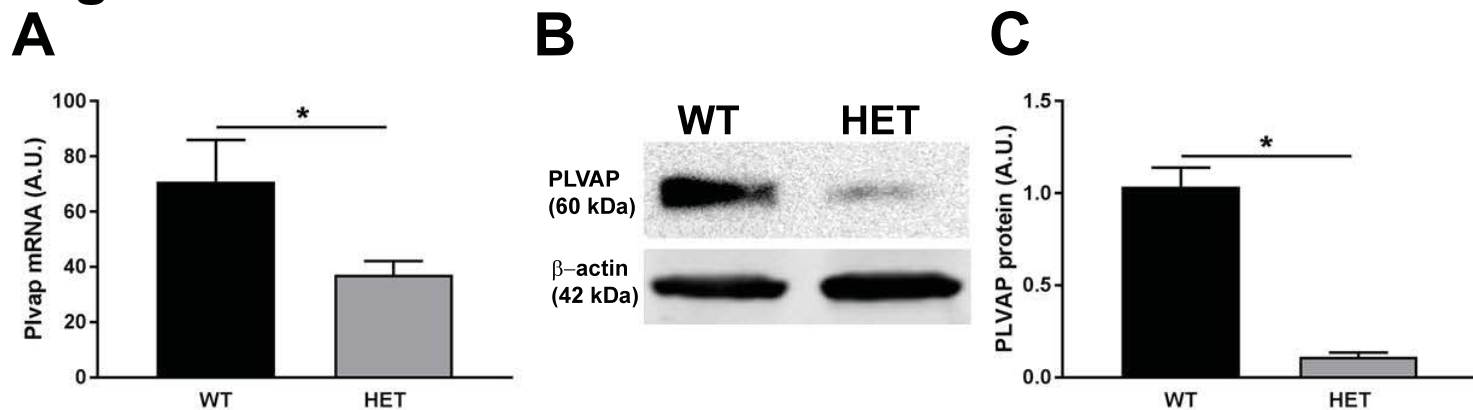**Figure S4.**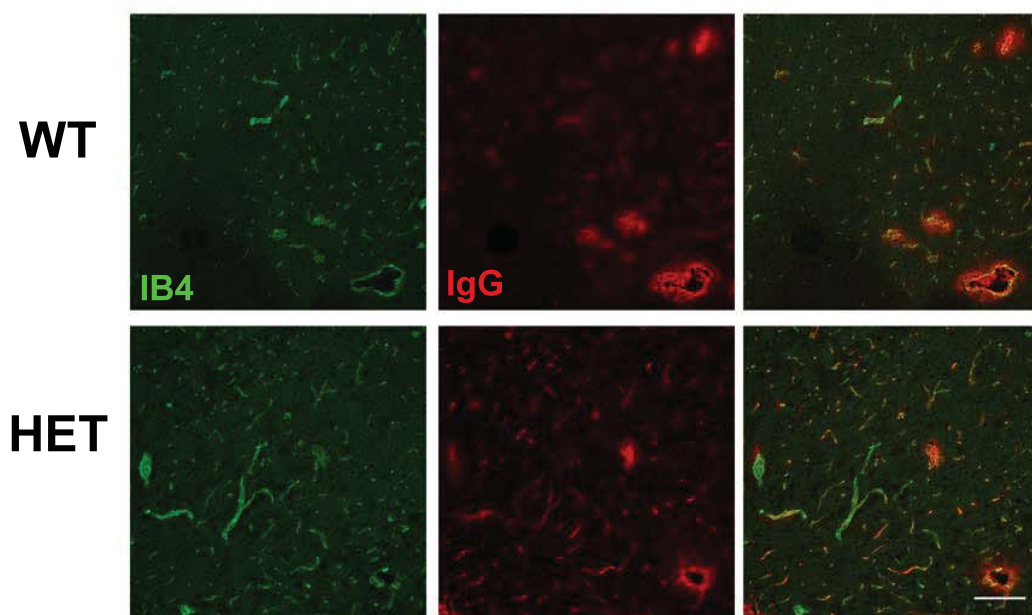**Figure S5.**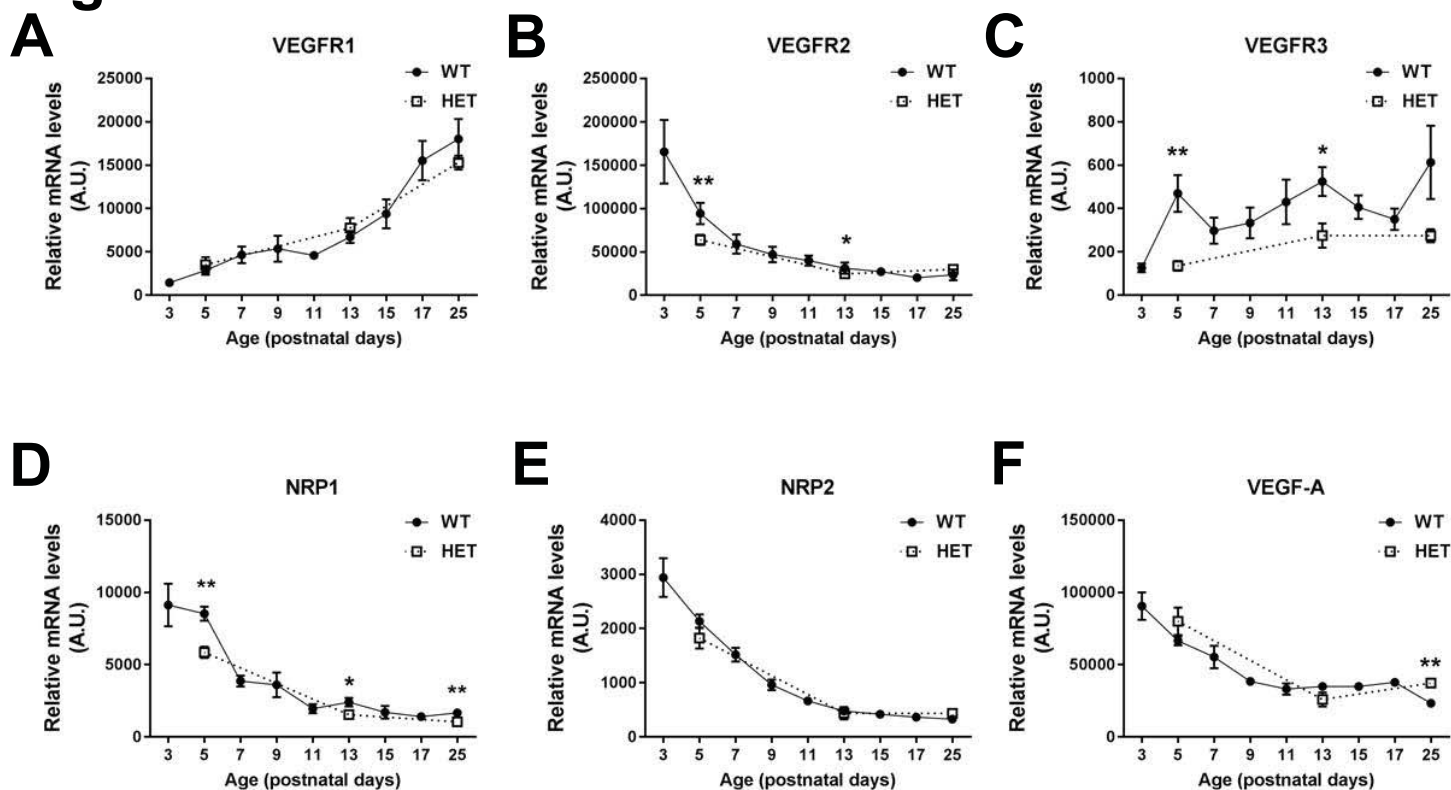

Supplement: Supplementary file 3 [file fj.201801499RRR.sf3.pdf]

**Figure S6.**

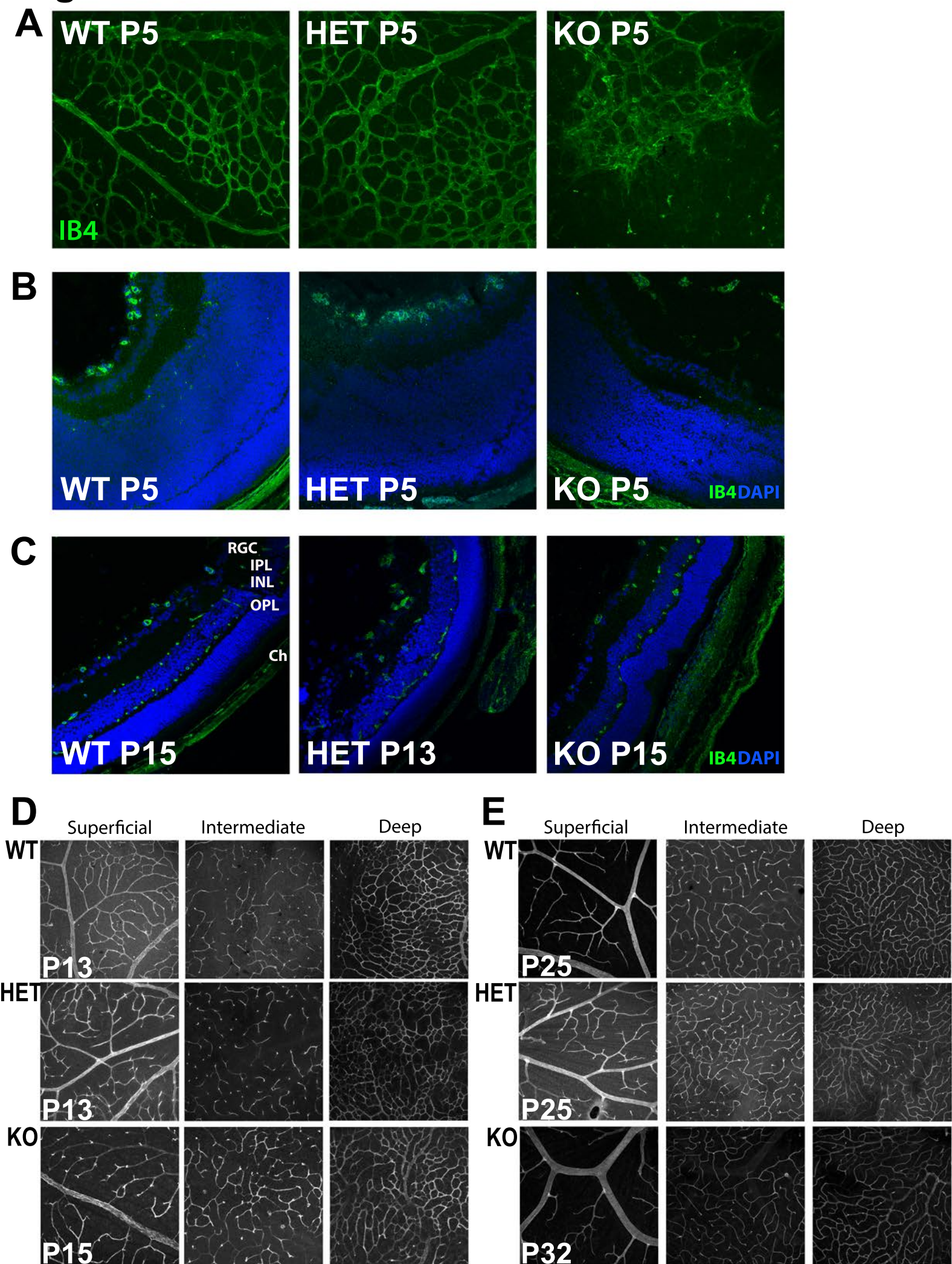

Supplement: Supplementary file 4 [file fj.201801499RRR.sf6.pdf]
