# Supplementary material for: Expression patterns of endothelial permeability pathways in the development of the blood-retinal barrier in mice
Source: FASEB J. 2019 Jan 30;33(4):5320–33. doi: 10.1096/fj.201801499RRR (PMC6436651; doi:10.1096/fj.201801499RRR)
Supplement: Supplementary file 7 [file fj.201801499RRR.sd1.doc]

**Supplemental information**

**Figure S1.** Leakage of the plasma protein IgG in the retina during BRB development. Retinal cryostat sections were stained for IgG (red), endothelial cells (isolectin B4; green) and nuclei (DAPI; blue). In the first postnatal weeks, leakage of IgG from the vessels occurred in the retinal ganglion cell layer, whereas at P15, IgG was confined to the lumen of blood vessels. RGC = retinal ganglion cell layer, IPL = inner plexiform layer, INL = inner nuclear layer, OPL = outer plexiform layer, ONL = outer nuclear layer, Ch = choriocapillaris. Scale bar = 100 µm.

**Figure S2.** Protein expression of claudin-5 **(A)**, ZO-1 **(B)** and VE-cadherin **(C)** in retinal cryostat sections was present at all ages. Scale bar = 50 µm. Isolectin B4 (IB4, green) was used to stain the retinal vessels and cell nuclei were stained with DAPI (blue). **(D)** Enlarged images of boxed regions of (A), (B) and (C). Brightness and contrast were linearly enhanced using ImageJ.

**Figure S3.** PLVAP expression in wildtype and *Plvap*^+/-^ mice. **(A)** *Plvap*^+/-^ mice (HET) showed significantly lower *Plvap* mRNA expression levels in kidney as compared to wildtype (WT) mice at P25. Also on the protein level, there was significantly less PLVAP expression in kidneys from HET mice, as compared to WT **(B)**. **(C)** Quantification of PLVAP expression from kidney samples of WT and HET mice at P29. *P<0.05. Data are depicted as mean±s.d.

**Figure S4.** Leakage of IgG (red) from blood vessels in the brain was similar in wildtype (WT) and *Plvap^+/-^* mice at P55. IB4 = isolectin B4 (green). Scale bar = 100 µm.

**Figure S5.** Expression of VEGFR1, -2, -3, NRP1, -2 and VEGF-A during retinal vascularization in wildtype (WT) and *Plvap*^+/-^ (HET) mice. mRNA levels of VEGFR1 **(A)**, VEGFR2 **(B)**, VEGFR3 **(C)**, NRP1 **(D)**, NRP2 **(E)** and VEGF-A **(F)** in the retinal vasculature of WT mice from P3 to P25; n=7-11 for all ages (closed circles), and HET mice at P5, P13 and P25; n=7-8 for both groups at each time point (open squares). *P<0.05, **P<0.01, difference between WT and HET at P5, P13 and P25. Data are depicted as mean±s.d.

**Figure S6.** Vascularization of the *Plvap*^-/-^ (KO) retina. Retinal vascularization at P5 was visualized in retinal wholemounts using isolectin B4 (green). In KO mice, the retinal vasculature at P5 was significantly delayed as compared to wildtype (WT) and *Plvap*^+/-^ (HET) mice **(A)**. Confocal images of a retinal cryostat section at P5 **(B)** and P13/15 **(C)** in WT, HET and KO mice. At P15, 3 parallel vascular layers were formed. Nuclei are stained with DAPI (blue) and vessels with IB4 (green). **(D)** At P13-15, the deep capillary plexus was connected, whereas the intermediate capillary plexus was still being generated in WT, HET and KO mice. **(E)** At P32, all 3 vascular plexi were formed and connected. The retinal vasculature was similar as in WT and HET mice (both P25 in this figure), although there may be less arteries, which was compensated for by more arterial branching in the central retina. IB4 = isolectin B4, RGC = retinal ganglion cell layer, IPL = inner plexiform layer, INL = inner nuclear layer, OPL = outer plexiform layer, Ch = choriocapillaris.

**Video S1.** Traversing retinal veins in the retina of a *Plvap*^+/-^ (HET) mouse at P30. A 3D image of the retinal vasculature was generated from a confocal z-stack of a retinal wholemount. The vasculature is depth coded (red = superficial vascular plexus, green/yellow = intermediate capillary layer, blue = deep capillary layer). The artery in the middle grows into the superficial vascular plexus, with capillaries branching off to generate the parallel intermediate and deep capillary layers. The 2 retinal veins (indicated by the white drawing pins) grow from the optic nerve head into the superficial vascular plexus, but traverse the retinal layers, ending in the deep capillary layer.

**Video S2. Retinal arteries and veins in the retina of a wildtype (WT) mouse at P25. A 3D image of the retinal vasculature was generated from a confocal z-stack of a retinal wholemount. The vasculature is depth coded (red = superficial vascular plexus, green/yellow = intermediate capillary layer, blue = deep capillary layer). The retinal artery and vein grow into the superficial vascular plexus and capillaries branch off to generate a parallel intermediate and deep capillary layer.**

|  |  | **Veins imaged** (n) | **Veins traversing** (n) | **Veins traversing** (% of imaged) | **Arteries imaged** (n) | **Arteries traversing** (n) | **Arteries traversing** (% of imaged) |
| --- | --- | --- | --- | --- | --- | --- | --- |
| **WT (n=14)** |  | 61 | 6 | 9.8 | 92 | 3 | 3.3 |
| **HET (n=17)** |  | 68 | 14 | 20.6 | 111 | 3 | 2.7 |

**Table S1.** Number of veins and arteries traversing from the superficial plexus to the deep capillary layer in retinas from wildtype (WT; n=14) and *Plvap*^+/-^ (HET; n=17) mice at P25-P35.

**Supplemental Methods**

*Macro for quantification of filopodia in the vascular sprouting front in ImageJ.*

/ *Input: Active closed ROI (e.g. wand-tool obtained closed loop or polygon selection)

* Action: click first point (S) of ROI to retain, click last point (E) of ROI to retain

* Output:Copy of input ROI in ROI manager on positon last-1.Polyline ROI of line segments connectin S with E along input ROI.Polyline ROI is active and is added to the ROI manager on position last.

*

* As part of the edge between two input points can be target for S or E, input ROI is interpolated. Interpolation by step 1 adds edge along each pixel side for each pixel, increasing length of original edge. Smoothing factor attempts to counter this error.Smoothing also counters fractal effect of background variation

*

* After first click (setting S) CW or CCW following the ROI contour is determined as soon as mouse moves

* After second click (setting E) the points ending up in the output ROI are determined by S, E and CW/CCW

*

* Log window shows coordinates and extra info if variable 'dbg' is set to 1.

* Known challenge: sometimes CW flips to CCW or vice versa when index boundary of input ROI is passed

*/

//

macro "Measure Part of ROI length"{

dbg=1; //1 shows debug information in the log window

//getCursorLoc(x,y,z,flags) flags:

// shift=1;

// ctrl=2;

// rightButton=4;

// alt=8;

// leftButton=16;

// insideROI = 32; // requires 1.42i or later

//

smoothing=5;

//roiManager("reset");

if(dbg==1) print("[\\Clear](../../../../%5C%5CClear)");mySelectionType = selectionType();

if (mySelectionType == -1){

print('no closed ROI selected'); return;

}

myRoi=roiManager("count");//array is zero based, so 1 element will be added in the next line

setColor('green');

run("Interpolate", 'interval='+smoothing);//5 is around 4 corners of 1 pixel

getSelectionCoordinates(xc,yc); roiManager('add'); roiManager("select", myRoi);

pStart=-1;

pEnd=-1;

walkingdirection=0;

r=7;d=2*r+1;

Overlay.show();

getCursorLoc(x1,y1,z,m1);

finished=false;

setColor('green');

do{

getCursorLoc(x2,y2,z,m2); x1=x2;y1=y2; n=nearest(x2,y2,xc,yc);

Overlay.remove; Overlay.drawEllipse(xc[n]-r,yc[n]-r,d,d); Overlay.show();

if(dbg==1) print("\\Update0:Point 1: cursor at"+n);

}while(m2&16==0);//wait for mouse button down

if(dbg==1) print ('point 1 set as '+n);

roiManager("select", myRoi);

do{

getCursorLoc(x1,y1,z,m1);//wait for mouse button up

} while(m1&16 !=0); pStart=n;

walkingdirection=0;showwalking=0;

do{

setColor('red');

Overlay.drawEllipse(xc[pStart]-r,yc[pStart]-r,d,d);

Overlay.add; Overlay.show();

setColor('green');

getCursorLoc(x2,y2,z,m2);

x1=x2;y1=y2; n=nearest(x2,y2,xc,yc);

//if n == pStart then we might need to change direction; walkingdirection=0;

if((abs(n-pStart) < 3)){//only flip walking direction when REAL CLOSE to the starting position

if(showwalking ==0 && walkingdirection==0){if(dbg==1) print("Walking pStart="+pStart); showwalking=1;}

if(n> pStart){//then we move CW;

walkingdirection=1;

if(dbg==1) print("\\Update1:Walking CW pStart="+pStart);

}

if(n< pStart){//then we move CCW;

walkingdirection=-1;

if(dbg==1) print("\\Update1:Walking CCW pStart="+pStart);

}

if(n== pStart) walkingdirection==0;//then we are where we started from

} //else we are too far away, do not change walking direction

if(dbg==1) print("\\Update2:Walking at"+n); //just update the position

Overlay.remove;

Overlay.drawEllipse(xc[n]-r,yc[n]-r,d,d);

Overlay.add;

Overlay.show();

}while (m2&16==0);

pEnd=n;

if(dbg==1) print('finished. Start is at '+pStart + " End at "+pEnd);

if (walkingdirection ==1){

//then CW

if(pStart < pEnd){ //walk from pStart to pEnd

subx=Array.slice(xc, pStart, pEnd);

suby=Array.slice(yc, pStart, pEnd);

if(dbg==1) print('pStart to pEnd');

}else{ //walk from pStart to length-1, 0 to bEnd

subx= Array.concat(

Array.slice(xc,pStart,xc.length-1),

Array.slice(xc,0,pEnd)

);

suby= Array.concat(

Array.slice(yc,pStart,xc.length-1),

Array.slice(yc,0,pEnd)

);

if(dbg==1) print('pStart,xc.length-1');

}

}else{

//CCW

if( pStart > pEnd){

subx=Array.slice(xc, pEnd, pStart);

suby=Array.slice(yc, pEnd, pStart);

if(dbg==1) print('pEnd, pStart');

}else{

subx= Array.concat(

Array.slice(xc,pEnd,xc.length-1),

Array.slice(xc,0,pStart)

);

suby= Array.concat(

Array.slice(yc,pEnd,xc.length-1),

Array.slice(yc,0,pStart)

);

if(dbg==1) print('pStart,xc.length-1 0,pEnd');

}

}

makeSelection("polyline",subx,suby);

run("Measure"); //!!

roiManager('add');

setColor('red');

Overlay.drawEllipse(xc[pStart]-r,yc[pStart]-r,d,d);

l=subx.length-1;

for(i=0;i<l;i++){

s=i; e=(s+1);

Overlay.drawLine(subx[s],suby[s], subx[e],suby[e]);

}

Overlay.add; Overlay.show();

roiManager("deselect"); run("Select None");

}

function nearest(x,y,cx,cy){

pp=0;

d=sqrt((cx[0]-x)*(cx[0]-x)+(cy[0]-y)*(cy[0]-y));//what coordinates(x,y) are closest to (x,y)?

for (i=1;i<cx.length;i++) {

di = sqrt((cx[i]-x)*(cx[i]-x)+(cy[i]-y)*(cy[i]-y));

if (di<d) {

d=di; pp=i;

}

}

return pp;

}
